# Supplementary material for: Multi‐dataset identification of innovative feature genes and molecular mechanisms in keratoconus
Source: J Cell Mol Med. 2024 Sep 19;28(18):e70079. doi: 10.1111/jcmm.70079 (PMC11412914; doi:10.1111/jcmm.70079)
Supplement: Supplementary file 2 — Material S2. [file JCMM-28-e70079-s002.zip › 7.GSEA analysis/4.MYRF/KEGG_CELL_CYCLE.html]

Details for gene set KEGG\_CELL\_CYCLE[GSEA]

|  || Dataset | MYRF.group.cls#h\_versus\_l |
| Phenotype | group.cls#h\_versus\_l |
| Upregulated in class | l |
| GeneSet | KEGG\_CELL\_CYCLE |
| Enrichment Score (ES) | -0.41598123 |
| Normalized Enrichment Score (NES) | -1.4436142 |
| Nominal p-value | 0.1301653 |
| FDR q-value | 0.20829591 |
| FWER p-Value | 0.83 |
Table: GSEA Results Summary

  

Fig 1: Enrichment plot: KEGG\_CELL\_CYCLE      
 Profile of the Running ES Score & Positions of GeneSet Members on the Rank Ordered List

  

| SYMBOL | TITLE | RANK IN GENE LIST | RANK METRIC SCORE | RUNNING ES | CORE ENRICHMENT || 1 | CCNE2 | na | 2609 | 0.317 | -0.0529 | No |
| 2 | CDC14A | na | 2729 | 0.310 | -0.0343 | No |
| 3 | PRKDC | na | 6379 | 0.181 | -0.1269 | No |
| 4 | ANAPC1 | na | 7351 | 0.154 | -0.1440 | No |
| 5 | ATM | na | 8036 | 0.138 | -0.1540 | No |
| 6 | ORC5 | na | 8199 | 0.133 | -0.1492 | No |
| 7 | SMC1A | na | 8302 | 0.131 | -0.1428 | No |
| 8 | CCNA1 | na | 9171 | 0.112 | -0.1599 | No |
| 9 | ATR | na | 9220 | 0.111 | -0.1535 | No |
| 10 | CREBBP | na | 9688 | 0.102 | -0.1597 | No |
| 11 | ESPL1 | na | 9710 | 0.101 | -0.1531 | No |
| 12 | GADD45G | na | 10627 | 0.084 | -0.1736 | No |
| 13 | CCNE1 | na | 11231 | 0.075 | -0.1857 | No |
| 14 | E2F3 | na | 11256 | 0.074 | -0.1812 | No |
| 15 | CDK1 | na | 11292 | 0.073 | -0.1770 | No |
| 16 | FZR1 | na | 11329 | 0.073 | -0.1728 | No |
| 17 | STAG2 | na | 11368 | 0.072 | -0.1688 | No |
| 18 | E2F2 | na | 12461 | 0.057 | -0.1963 | No |
| 19 | STAG1 | na | 12552 | 0.056 | -0.1949 | No |
| 20 | CDC23 | na | 12691 | 0.054 | -0.1951 | No |
| 21 | RB1 | na | 12759 | 0.053 | -0.1933 | No |
| 22 | TP53 | na | 12977 | 0.051 | -0.1959 | No |
| 23 | SMAD4 | na | 13356 | 0.045 | -0.2036 | No |
| 24 | YWHAB | na | 13423 | 0.045 | -0.2024 | No |
| 25 | MDM2 | na | 13578 | 0.043 | -0.2038 | No |
| 26 | BUB3 | na | 13749 | 0.041 | -0.2058 | No |
| 27 | ANAPC7 | na | 13808 | 0.040 | -0.2046 | No |
| 28 | CDKN1B | na | 14078 | 0.037 | -0.2098 | No |
| 29 | ORC2 | na | 14201 | 0.036 | -0.2108 | No |
| 30 | CCND1 | na | 14657 | 0.031 | -0.2217 | No |
| 31 | RBX1 | na | 14789 | 0.030 | -0.2234 | No |
| 32 | SMC1B | na | 15002 | 0.028 | -0.2275 | No |
| 33 | ANAPC13 | na | 15453 | 0.023 | -0.2389 | No |
| 34 | ORC4 | na | 15655 | 0.021 | -0.2432 | No |
| 35 | YWHAZ | na | 16350 | 0.014 | -0.2622 | No |
| 36 | ANAPC4 | na | 16590 | 0.012 | -0.2683 | No |
| 37 | YWHAE | na | 16676 | 0.011 | -0.2699 | No |
| 38 | SKP1 | na | 17295 | 0.006 | -0.2874 | No |
| 39 | ANAPC11 | na | 17415 | 0.005 | -0.2905 | No |
| 40 | TFDP1 | na | 17568 | 0.004 | -0.2946 | No |
| 41 | SMAD3 | na | 17747 | 0.002 | -0.2996 | No |
| 42 | RBL1 | na | 17757 | 0.002 | -0.2997 | No |
| 43 | HDAC2 | na | 17814 | 0.001 | -0.3013 | No |
| 44 | RBL2 | na | 18064 | -0.001 | -0.3084 | No |
| 45 | SMAD2 | na | 18091 | -0.001 | -0.3091 | No |
| 46 | PTTG2 | na | 18336 | -0.004 | -0.3158 | No |
| 47 | ANAPC5 | na | 18440 | -0.005 | -0.3185 | No |
| 48 | PCNA | na | 18478 | -0.005 | -0.3192 | No |
| 49 | CDKN2D | na | 18581 | -0.006 | -0.3217 | No |
| 50 | PTTG1 | na | 19038 | -0.011 | -0.3341 | No |
| 51 | CDC16 | na | 19192 | -0.013 | -0.3377 | No |
| 52 | GSK3B | na | 19466 | -0.016 | -0.3444 | No |
| 53 | HDAC1 | na | 19552 | -0.017 | -0.3457 | No |
| 54 | CDC27 | na | 19757 | -0.019 | -0.3503 | No |
| 55 | CDKN2A | na | 19779 | -0.019 | -0.3495 | No |
| 56 | CDC14B | na | 20136 | -0.024 | -0.3581 | No |
| 57 | YWHAG | na | 20695 | -0.030 | -0.3721 | No |
| 58 | CUL1 | na | 21468 | -0.039 | -0.3917 | No |
| 59 | MCM3 | na | 21484 | -0.039 | -0.3893 | No |
| 60 | E2F1 | na | 21781 | -0.043 | -0.3948 | No |
| 61 | CDKN2B | na | 21823 | -0.044 | -0.3929 | No |
| 62 | RAD21 | na | 21935 | -0.045 | -0.3929 | No |
| 63 | ORC3 | na | 21973 | -0.045 | -0.3908 | No |
| 64 | TGFB2 | na | 22177 | -0.048 | -0.3932 | No |
| 65 | CCNH | na | 22182 | -0.048 | -0.3899 | No |
| 66 | MAD2L1 | na | 22188 | -0.048 | -0.3867 | No |
| 67 | SKP2 | na | 22430 | -0.051 | -0.3900 | No |
| 68 | TFDP2 | na | 22590 | -0.053 | -0.3908 | No |
| 69 | YWHAQ | na | 22775 | -0.056 | -0.3921 | No |
| 70 | SFN | na | 22799 | -0.057 | -0.3888 | No |
| 71 | CCND3 | na | 23579 | -0.069 | -0.4064 | No |
| 72 | ABL1 | na | 23912 | -0.074 | -0.4107 | Yes |
| 73 | CDC26 | na | 23958 | -0.075 | -0.4067 | Yes |
| 74 | ANAPC10 | na | 24062 | -0.077 | -0.4042 | Yes |
| 75 | DBF4 | na | 24067 | -0.077 | -0.3989 | Yes |
| 76 | MCM7 | na | 24141 | -0.078 | -0.3954 | Yes |
| 77 | CDC7 | na | 24203 | -0.079 | -0.3916 | Yes |
| 78 | ANAPC2 | na | 24204 | -0.079 | -0.3860 | Yes |
| 79 | CCNB3 | na | 24284 | -0.080 | -0.3825 | Yes |
| 80 | CDKN2C | na | 24579 | -0.085 | -0.3850 | Yes |
| 81 | E2F5 | na | 24596 | -0.086 | -0.3793 | Yes |
| 82 | CDK7 | na | 24636 | -0.087 | -0.3743 | Yes |
| 83 | CDK4 | na | 24674 | -0.088 | -0.3692 | Yes |
| 84 | CCNB2 | na | 25069 | -0.094 | -0.3738 | Yes |
| 85 | CDC20 | na | 25083 | -0.095 | -0.3675 | Yes |
| 86 | CDK6 | na | 25789 | -0.108 | -0.3802 | Yes |
| 87 | CCND2 | na | 25792 | -0.109 | -0.3725 | Yes |
| 88 | SMC3 | na | 25918 | -0.111 | -0.3683 | Yes |
| 89 | BUB1 | na | 26277 | -0.119 | -0.3702 | Yes |
| 90 | CCNB1 | na | 26279 | -0.119 | -0.3618 | Yes |
| 91 | MAD1L1 | na | 26280 | -0.119 | -0.3533 | Yes |
| 92 | TTK | na | 26386 | -0.121 | -0.3478 | Yes |
| 93 | MCM4 | na | 26605 | -0.126 | -0.3452 | Yes |
| 94 | PKMYT1 | na | 26932 | -0.134 | -0.3451 | Yes |
| 95 | MAD2L2 | na | 27045 | -0.136 | -0.3387 | Yes |
| 96 | EP300 | na | 27396 | -0.145 | -0.3385 | Yes |
| 97 | CHEK2 | na | 27601 | -0.151 | -0.3337 | Yes |
| 98 | MYC | na | 27707 | -0.153 | -0.3259 | Yes |
| 99 | CDC25A | na | 27710 | -0.153 | -0.3151 | Yes |
| 100 | GADD45B | na | 27736 | -0.154 | -0.3049 | Yes |
| 101 | ZBTB17 | na | 28296 | -0.170 | -0.3090 | Yes |
| 102 | CDC25B | na | 28392 | -0.172 | -0.2995 | Yes |
| 103 | MCM5 | na | 28412 | -0.173 | -0.2878 | Yes |
| 104 | CCNA2 | na | 28614 | -0.178 | -0.2810 | Yes |
| 105 | MCM6 | na | 29019 | -0.192 | -0.2790 | Yes |
| 106 | CDK2 | na | 29418 | -0.203 | -0.2761 | Yes |
| 107 | E2F4 | na | 30051 | -0.225 | -0.2784 | Yes |
| 108 | MCM2 | na | 30505 | -0.242 | -0.2743 | Yes |
| 109 | WEE2 | na | 30984 | -0.263 | -0.2694 | Yes |
| 110 | CHEK1 | na | 31328 | -0.281 | -0.2594 | Yes |
| 111 | CDC45 | na | 31350 | -0.282 | -0.2400 | Yes |
| 112 | YWHAH | na | 31453 | -0.288 | -0.2224 | Yes |
| 113 | WEE1 | na | 31710 | -0.302 | -0.2084 | Yes |
| 114 | CDKN1C | na | 31787 | -0.306 | -0.1889 | Yes |
| 115 | CDC6 | na | 31874 | -0.310 | -0.1693 | Yes |
| 116 | CDC25C | na | 31922 | -0.313 | -0.1485 | Yes |
| 117 | PLK1 | na | 32621 | -0.359 | -0.1431 | Yes |
| 118 | ORC6 | na | 32672 | -0.363 | -0.1188 | Yes |
| 119 | GADD45A | na | 32736 | -0.368 | -0.0945 | Yes |
| 120 | TGFB1 | na | 32868 | -0.377 | -0.0716 | Yes |
| 121 | TGFB3 | na | 33024 | -0.389 | -0.0484 | Yes |
| 122 | BUB1B | na | 33183 | -0.405 | -0.0242 | Yes |
| 123 | CDKN1A | na | 33771 | -0.483 | -0.0069 | Yes |
| 124 | ORC1 | na | 33804 | -0.486 | 0.0267 | Yes |
Table: GSEA details [plain text format]

  

Fig 2: KEGG\_CELL\_CYCLE      
 Blue-Pink O' Gram in the Space of the Analyzed GeneSet

  

Fig 3: KEGG\_CELL\_CYCLE: Random ES distribution      
 Gene set null distribution of ES for **KEGG\_CELL\_CYCLE**
